# Plant Extract in the Control of Poultry Omphalitis

**DOI:** 10.3390/pathogens13060438

**Published:** 2024-05-23

**Authors:** Gabriel da Silva Oliveira, Paula Gabriela da Silva Pires, Concepta McManus, Luana Maria de Jesus, Pedro Henrique Gomes de Sá Santos, Vinícius Machado dos Santos

**Affiliations:** 1 Faculty of Agronomy and Veterinary Medicine, University of Brasília, Brasília 70910-900, Brazil; gabriels.unb@gmail.com (G.d.S.O.);; 2 Animal Production and Health Postgraduate Program, Catarinense Federal Institute—Campus Concórdia, Concórdia 89703-720, Brazil; 3 Laboratory of Poultry Science, Federal Institute of Brasília—Campus Planaltina, Brasília 73380-900, Brazil

**Keywords:** antibacterials, microorganisms, natural products, poultry infections, sanitizers

## Abstract

Bacteria continue to disrupt poultry production and can cause resistant and persistent yolk sac infections to prevention efforts, known as omphalitis, resulting in poultry death. This literature review aims to demonstrate how plant extracts can help combat omphalitis in poultry. The Google Scholar database served as a resource for retrieving pertinent literature covering a wide range of search terms relevant to the scope of the research. The search strategy involved a combination of terms such as antimicrobials, chick embryo, omphalitis, plant extracts, poultry nutrition, and sanitization. The potential of plant extracts in preventing or treating infections in poultry, especially omphalitis, is mainly due to their antibacterial and safety properties. Sanitization and direct delivery of plant extracts to the internal contents of eggs, feed, or water are cutting-edge interventions to reduce the bacterial load in eggs and poultry, minimizing infection rates. For example, these interventions may include advanced treatment technologies or precise delivery systems focused on disease prevention in poultry.

## 1. Introduction

**Figure 1:**
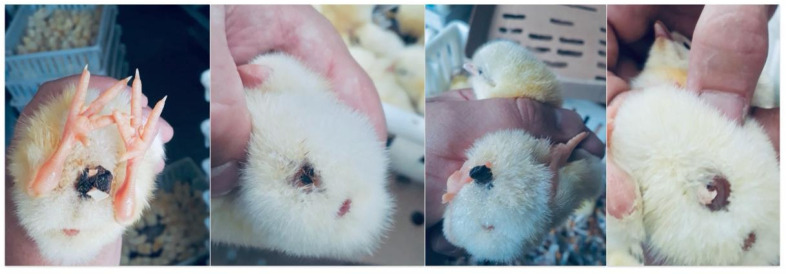
Chicks omphalitis diagnosis.

The eggshell has a significant relevance in the contamination of poultry, as it can contain a considerable count of mesophilic bacteria and enterobacteria. Bacterial infections can affect poultry in two main ways. The first is through direct infection of the embryo, which occurs when bacteria capable of penetrating the eggshell manage to infect it. The second method occurs through bacteria that, although they cannot penetrate the shell, can infect poultry shortly after hatching. Both forms can lead to systemic infection, including that of the yolk sac (Figure 1). This infectious condition, known as omphalitis, is characterized by incomplete closure of the navel after insertion of the yolk sac into the abdominal cavity and is recognized as the main reason for the decreased survival rates of newly arrived chicks in the poultry sheds [1]. Azevedo et al. [2] conducted a study to evaluate the causes of mortality of 371,997 poultry. The results of the assessments revealed that around 26,040 poultry died due to omphalitis. This finding implies significant economic losses for poultry producers. The financial ramifications of omphalitis include additional labor costs for prevention and treatment and changes in the quantity of poultry products sold [3].

Poultry farms dedicated to producing meat and eggs constitute favorable environments for the proliferation of opportunistic microorganisms. Various microbial agents have been identified in such environments, demonstrating significant variability between farms targeted for different purposes. On meat farms, a rigorous analysis revealed a remarkable composition of the opportunistic microbiota, with the following protagonists highlighted: *Enterococcus faecium* (32.2%), *Staphylococcus aureus* (30.6%), *Pseudomonas aeruginosa* (18.6%), *Staphylococcus epidermidis* (5.2%), *Candida albicans* (4.7%), *Proteus* spp. (2.8%), *Aspergillus* spp. (2.6%), and *Escherichia coli* (2.2%). Additional microorganisms, such as *Staphylococcus saprophyticus*, *Enterobacter* spp., and other species, comprised a minority fraction, totaling less than 1% of the microbial spectrum [4]. On farms focused on egg production, a distinct distribution emerged, with notable variation in the detection frequency of different strains. Here, microbiological leaders included *E. faecium* (23.9%), *P. aeruginosa* (19.7%), *E. coli* (13.9%), *S. aureus* (11.4%), *Aspergillus* spp. (9.3%), *S. epidermidis* (9.2%), *Enterobacter* spp. (8.9%), *C. albicans* (2.7%), *Proteus* spp. (0.5%), and a residual fraction of other microorganisms, contributing less than 0.5% of the total composition [4]. Among the microorganisms identified, *E. coli* stands out as a prominent figure, assuming the role of the leading causal agent of omphalitis in poultry [5,6]. These opportunistic microorganisms pose a substantial threat to poultry health, especially when poultry have compromised immune systems. Increasing multidrug resistance among bacterial strains affecting poultry with omphalitis is rising in the poultry sector. Notably, *E. coli* strains isolated from chicks with omphalitis, despite exhibiting high sensitivity to meropenem, gentamicin, colistin, and kanamycin, paradoxically manifest significant resistance to essential antibiotics such as erythromycin, oxacillin, clindamycin, nalidixic acid, and ampicillin [6]. In another study by Mohibbullah et al. [5], *E. coli* isolates from chicks with omphalitis also exhibited worrying resistance profiles. These strains have demonstrated antibiotic resistance, including erythromycin, amoxicillin, tetracycline, ampicillin, and cefixime.

The prevention and treatment of omphalitis can occur through poultry practices that include the immunization and individualized assessment of poultry, sanitization, feed and water supplementation, asepsis of sheds and hatcheries, and strict waste management. Plant extract sanitizers can significantly reduce the number of bacteria on hatching egg shells [7]. The administration of plant extracts in ovo and supplied through the diet or water can improve poultry immunity [8,9,10]. These extracts, which can be used to combat omphalitis, are alternatives to prevent and reduce infection and avian mortality and do not generate bacterial resistance [11]. In research led by Oskay et al. [12], the ability of *E. coli* to resist the antibiotics ampicillin, amoxicillin, and sulfamethoxazole was overcome using extracts from eight plants. *E. coli* demonstrated sensitivity to a relatively low concentration (4 mg/mL) of extracts from *Punica granatum* (Pomegranate), *Conyza canadensis* (Canadian fleabane), *Euphorbia peplus* (Petty spurge), *Citrus reticulata* (Mandarin), *Liquidambar orientalis* (Sweetgum), *Vitis vinifera* (Grapevine), *Rosmarinus officinalis* (Rosemary), and *Ecballium elaterium* (Squirting cucumber). The disk diffusion technique revealed inhibition halos ranging from 8 to 16 mm around the disks soaked with these extracts. In addition to suggesting the antibacterial efficacy of the extracts, this phenomenon shows their potential use in the battle against resistant strains of *E. coli.* This may be economically viable, as the plant extracts are effective at relatively low concentrations. The acquisition cost and antibacterial efficacy are relevant in this case.

This literature review aims to demonstrate how plant extracts can help combat omphalitis in poultry.

## 2. Methodology Used to Select the Studies Used in Subsequent Topics

### 2.1. Search Mechanisms

To identify potentially relevant publications, we searched Google Scholar using the following search terms combined or alone: antimicrobials, chick embryo, omphalitis, chicks, plant extracts, poultry, broilers, laying hens, hatching eggs, eggshells, sanitization, in ovo injection, poultry nutrition, poultry infection, egg contamination, and poultry contamination. We did not limit the publication period, but the selected publications had to be written in English or Portuguese. We used the previously mentioned terms to write each subsequent topic and conducted two different Portuguese and English searches. We then selected the first 10 results of each search for analysis. Searches were initially performed on 1 March 2024, with subsequent updates conducted on 3 April 2024 and 20 April 2024, and finally on 15 May 2024.

### 2.2. Eligibility Criteria

Original articles, reviews, conference papers, and book chapters published in English or Portuguese that addressed the related search terms, focusing on avian omphalitis, were included. Studies that investigated the use of essential oils to treat avian omphalitis were excluded. Furthermore, studies in languages other than English or Portuguese were excluded. Articles that did not have the full text were also excluded. No exclusion criteria related to the poultry species were established. No specific criteria were established for the type of plant extract used. However, studies in which the plant species that originated the extracts were not identified were considered inappropriate for this analysis.

### 2.3. Screening and Data Extraction

All publications obtained in the search were imported into Mendeley. After removing duplicates, publications were evaluated by title and abstract. Next, the full texts of the selected publications were reviewed to ensure the material’s relevance and reliability before extracting the information necessary to write this review. They were imported into Excel (Microsoft 365, version 2404) to extract data from all eligible studies. Each included study was thoroughly analyzed, and the information was standardized.

Initially, 40 publications could have been considered for each topic. However, after a rigorous analysis of the eligibility criteria, only 8 publications were selected for the topic “Poultry omphalitis”, 11 for “Rapid Intervention in Omphalitis Infection to Minimize Bacterial Transmission,” 28 for “Plant Extracts”, and 15 for “Regimens based on plant extracts to control omphalitis”. During the review process, six publications were excluded from the topic “Poultry Omphalitis”, four from “Rapid Intervention in Omphalitis Infection to Minimize Bacterial Transmission”, four from “Plant Extracts”, and two from “Regimens based on plant extracts to control omphalitis”, as each paragraph reached its ideal conclusion. Thus, in the end, 2 publications were reviewed for “Poultry omphalitis”, 7 for “Rapid intervention in omphalitis infection to minimize bacterial transmission”, 24 for “Plant extracts”, and 13 for “Plant extract-based regimens to control omphalitis”.

## 3. Poultry Omphalitis

Omphalitis is a potentially fatal condition for poultry, characterized by a multisystemic infection resulting from an intense response to yolk sac infection that extends deep into the animal. The spectrum of infection can range from asymptomatic infections and moderate signs to more serious complications such as septicemia [13]. Putrefactive odor and a distended and soft abdomen with an inflamed, thickened, and spoiled vent region are described as characteristic clinical signs of yolk sac infection [14]. The survival of chicks for several days after infection suggests potential systemic dissemination of the infectious agent [14]. The method currently considered for diagnosing omphalitis is clinical observation when the chicks are selected immediately after hatching if the signs are already visible in the hatchery or the first week of the chicks’ life on the farm. Microbiological analysis of the yolk sac is also recommended to identify the causative bacteria.

Poultry systems are fertile environments for contamination, including the persistence of difficult-to-treat microorganisms and poultry susceptible to infections. The risk factors that favor the development of poultry infections such as omphalitis can be triggered by three distinct stages: pre-incubation (before the eggs are artificially incubated), incubation handling (during egg incubation), and post-hatching (after chicks hatch) (Table 1). Sources of contamination can be as primary as the handler’s hand, tray, countertops, drinking fountains, feeders, and clothing, among others.

## 4. Rapid Intervention of Omphalitis Infection to Minimize Bacterial Transmission

Health intervention in poultry farming must include a detailed assessment of the contamination history of the farm and hatcheries, as well as potential associated risk factors. Identifying and addressing microbiological disorders, such as omphalitis, is crucial. The most effective strategy against omphalitis is to prevent its occurrence in poultry, ensure adequate healing of the navel, and promote good absorption of the residual yolk sac. The chick quality assessment system categorizes chicks into two groups: chicks with healed or non-healed navels [15]. In addition to compromising the poultry quality of life, the latter makes housing them in the shed unfeasible, as most of the time, it will result in mortality [16]. Therefore, all efforts of professionals working in poultry farming are to prevent the presence of bacterial inoculums in the poultry environment, especially eggs and the poultry themselves. Sanitization and the direct supply of antimicrobials to the internal contents of eggs, feed, or water are cutting-edge interventions to reduce the bacterial load in eggs and poultry [17,18,19,20,21]. These practices typically kill contaminating bacteria and prevent the formation of biofilms that are devastating to poultry. The success rates of sanitization practices, in ovo delivery, and feed and water supplementation with antimicrobials can be high, and the recurrence rates of contamination can be low [17,18,19,20,21].

## 5. Plant Extracts

Plant extracts, green chemicals with high antimicrobial potential, have been tested for decades as feed additives, sanitizers, and food preservatives in poultry farming [7,22,23,24,25]. Furthermore, they were tested as personal hygiene products [26]. Chemical analysis of plant extracts typically detects the presence of alkaloids, flavonoids, terpenoids, steroids, phenolic compounds, tannins, and several other phytoconstituents [27]. These phytocompounds in plant extracts make their industrial use viable, including pharmaceutical, food, cosmetic, cleaning and sanitizing, agricultural, livestock, and personal hygiene sectors. Extracts can be extracted from plants by maceration, percolation, Soxhlet extraction, decoction, and hydrodistillation using solvents such as water, ethanol, methanol, chloroform, dichloromethanol, ether, and acetone [28].

Several standard or isolated bacteria are sensitive to plant extracts. An in vitro study aimed to evaluate, using the disk diffusion method, the effect of 20 μL of *Eugenia caryophyllata* (Clove) extract against some pathogenic bacterial strains and reported that the *E. caryophyllata* extract had the power to inhibit the growth of *Bacillus cereus* and *Salmonella* spp. [29]. Danish et. al. [30] analyzed the antibacterial effect of *Aloe vera* (Aloe) extract at doses of 10, 20, 25, and 30 μL. The results obtained by the disk diffusion method showed that among the bacteria sensitive to the extracts are *Bacillus subtilis*, *Bacillus cereus*, *Streptococcus pyogenes*, *S. aureus*, *E. coli*, *Enterococcus faecalis*, *P. aeruginosa*, and *Salmonella* Typhi. One study investigated the antibacterial profile of 10 plant extracts per broth microdilution technique. Of the extracts tested (*Arctium lappa* (Burdock), *Tanacetum vulgare* (Tansy), *Erythrina speciosa* (Brazilian coral tree), *Psidium guajava* (Guava), *Mikania glomerata* (Guaco), *Piper regnellii* (Red Piper), *Eugenia uniflora* (Surinam cherry), *P. granatum*, *Sambucus canadensis* (Elderberry), and *Plantago major* (Plantain)), seven showed some activity against *E. coli*, *P. aeruginosa*, *Bacillus subtilis*, or *S. aureus* at concentrations equal to or less than 1000 µg/mL. *P. regnellii* extract stood out as the most effective, inhibiting the growth of all bacteria tested [31]. Years later, Eller et al. [32] evaluated the in vitro antibacterial activity of six plant extracts using the disk diffusion method: *Anacardium occidentale* (Cashew), *Stryphnodendron adstringens* (Barbatimão), *Myracrodruon urundeuva* (Aroeira), *Cnidoscolus phyllacanthus* (Favela), *Heliotropium indicum* (Indian heliotrope), and *Sideroxylon obtusifolium* (Quixaba). Using 20 μL of extracts from *A. occidentale*, *S. adstringens*, or *M. urundeuva*, inhibition of the growth of *S. aureus* was observed. In line with the studies mentioned above, Mostafa et al. [33] investigated the effect of extracts of *Cuminum cyminum* (Cumin), *P. granatum*, *Syzygium aromaticum* (Clove), *Zingiber officinales* (Ginger), and *Thymus vulgaris* (Thyme) at a concentration of 10 mg/mL against *Bacillus cereus*, *S. aureus*, *E. coli*, *P. aeruginosa*, and *S. typhi*. They revealed that all extracts showed antibacterial efficiency against at least one bacteria tested by the disk diffusion method.

As mentioned previously, *E. coli* is the main bacteria associated with omphalitis. Interestingly, *E. coli* of avian origin has demonstrated sensitivity to several plant extracts (Table 2). It has been documented that plant extracts containing flavonoids, anthraquinones, alkaloids, tannins, phenolic compounds, and saponins have potent antibacterial activities [34]. Plant extracts can induce bacterial death by acting on several targets in the bacterial cell. Due to their affinity with bacterial membranes, plant extracts cause their permeabilization, which is the main target for bacterial eradication [35]. This mechanism can cause coagulation of bacterial protoplasm, inhibiting its growth [36]. The results presented by Bandian et al. [37] echo and strengthen the findings mentioned above, highlighting the assertive action of plant extracts on bacteria. These compounds trigger a process of degeneration of cell walls, culminating in the exudation of cytoplasmic material.

Extracts made from the plant species mentioned in this section demonstrate viability and significant potential for application in the control and prevention of omphalitis. This potential is attributed to the chemical composition of these extracts, which have robust antibacterial properties. The presence of compounds such as phenolic acids, including caffeic, gallic and ellagic acids, as well as flavonoids, including kaempferol, quercetin and rutin, can characterize the antibacterial activity of plant extracts [44]. Developing antibiotic plans using plant extracts deserves a comprehensive analysis as an alternative to traditional antibiotics in poultry farming. In this context, we consider some specific antibacterial protocols, discussed below, outlining possible applications to control avian omphalitis.

## 6. Regimens Based on Plant Extracts to Control Omphalitis

Given the high rate of poultry mortality due to omphalitis and the growing trend of bacterial contamination of poultry due to the high production flow of meat and layer poultry farming, antibiotic therapy emerges as a measure to achieve successful, productive results. However, it is expected that synthetic antibiotic therapy will no longer be the priority protocol in the future. To date, poultry farming still depends on synthetic chemical antibiotics to guarantee a good rate and productive quality of meat and eggs, satisfying the dietary needs of the global population. Despite this, inadequate or repetitive management of synthetic chemical antibiotics can result in water and soil pollution and threaten food safety and human and animal health [45,46].

Antibacterial protocols currently of interest in poultry farming include using plant extracts. These are plant-derived antibiotic compounds available for inclusion in green health programs. These programs involve using environmentally friendly compounds and methodologies to control and monitor microbiological problems in poultry farming sustainably. Systemic plant extracts can be targeted for the prevention and treatment of avian infections, especially in inaccessible locations, when topical treatment is not effective or visible and accessible infections that do not respond adequately to topical treatments. However, a paramount concern arises regarding potential side effects, considering that such substances are distributed through the poultry circulatory system after ingestion. Some plant extracts are viewed from the point of view of their potential to support control bacterial infections in poultry without adverse effects that compromise their survival. These extracts can come from (1) *Quercus infectoria* (Oak galls), *P. granatum* and *Senna alexandrina* (Senna) [47], (2) *P. granatum* [48], (3) Eucalyptus [7], (4) *T. vulgaris*, *Cinnamomum cassia* (Cinnamon) and *S. aromaticum* [49], (5) *Moringa oleifera* (Moringa) [9], (6) *P. guajava* [22], (7) *Camellia oleífera* (Tea seed) [8], (8) *C.sativa* and *S. aromaticum* [40], (9) *Tinospora cordifolia* (Guduchi) [50], and (10) *Azadirachta indica* (Neem) [10] (Table 3). *Citrus aurantifolia* (Tahiti lemon) and *Ocimum basilicum* (Basil) are also plants that can be used as a base for formulating antibacterial extracts for control applications in poultry farming [51].

**Figure 2:**
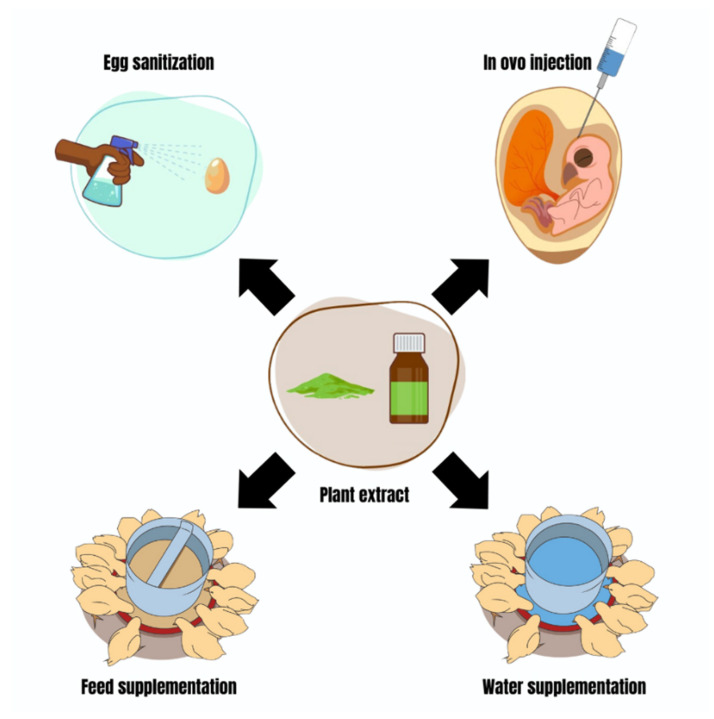
Routes of application of plant extracts for microbial control of omphalitis in poultry.

As an alternative to conventional antibiotic therapy, plant extracts have shown promise in preventing and treating infections in poultry farming, especially omphalitis. This is mainly due to their antibacterial activity and ability to prevent or treat infections in an economically viable way with minimal disruption to poultry health. Studies have investigated developing embryos and poultry after hatching against infections, using antibacterial protocols with plant extracts. These studies employed egg sanitization programs, in ovo injection techniques, and feed or water supplementation after hatching (Table 3; Figure 2). These studies showed that control programs using different methods resulted in significant benefits, such as effective bacterial control, improved health, and minimization of aggressive damage that could lead to the death of poultry. This shows that green antibiotic therapy has the potential to revolutionize poultry health management, seeking to maintain this production chain in an increasingly ecological context and being concerned with animal, human, and environmental health in parallel with good productivity. It is essential to highlight that the characteristics inherent to the plants, concentration, volume, toxicity, and the responses after application of the extract may vary for each protocol. Therefore, in-depth analysis is essential before commercial implementation on a large or small scale.

## 7. Conclusions

Given the seriousness of the issue, researchers in the poultry field have developed therapeutic strategies for preventing and treating infections in poultry based on green therapies using so-called plant extracts. These extracts are solutions obtained from different parts of plants and, according to the results obtained so far, have demonstrated significant success in combating avian infections. In the search for explored strategies to combat bacterial infections, such as avian omphalitis, a notable example is the use of extract derived from *S. aromaticum*. The administration of plant extracts to poultry, whether directly or indirectly, has been associated with lower production losses and improved poultry life quality. The present review may encourage researchers to deepen the existing findings on developing plant extracts to prevent or treat poultry infections. The three main challenges facing using plant extracts as antibacterial agents in poultry farming are outlined below:

- Precisely determining the total cost associated with obtaining and applying plant extracts in the poultry farming context remains uncertain.
- The availability of the plant species required for the production of antibacterial extracts.
- The lack of a solid scientific basis that clearly delineates the toxicity profile of plant extracts, including those that are widespread.

## Figures and Tables

**Table 1:** Some factors that promote poultry infection.

Risk Factors For Poultry Infection		
Pre-Incubation	Incubation Handling	Post-Hatching
Sanitary and environmental deficiencies in poultry sheds	Inadequate physical parameters of incubation	Sanitary and environmental deficiencies in poultry sheds
Improper egg handling	Failure to remove infertile eggs	Absence of vaccination
Contaminated breeders	Inadequately sanitized setters	Inadequate chick handling

**Table 2:** Some plant extracts efficient against bacteria of avian origin.

Study	Plant Extract (Common Name)	Plant Extract (Scientific Name)	Extraction Solvent	Sensitive Bacteria
[38]	Lemon Balm	*Melissa officinalis*	Water/ethyl alcohol	*E. coli*
	Baru Nut	*Dipteryx alata*	Water/ethyl alcohol	*E. coli*
	Aloe	*A. vera*	Water/ethyl alcohol	*E. coli*
	Lemongrass	*Cymbopogon citratrus*	Water/ethyl alcohol	*E. coli*
	Peppermint	*Mentha spicata*	Water/ethyl alcohol	*E. coli*
	Wormseed	*Chenopodium ambrosioides*	Water/ethyl alcohol	*E. coli*
	Wormwood	*Artemisia absinthium*	Water/ethyl alcohol	*E. coli*
	Madagascar Periwinkle	*Catharanthus roseus*	Water/ethyl alcohol	*E. coli*
	Plantain	*P. major*	Water/ethyl alcohol	*E. coli*
	Scotch Bonnet Pepper	*Capsicum chinense*	Water/ethyl alcohol	*E. coli*
[39]	Spike Moss	*Selaginella involvens*	Petroleum ether/benzene/methanol/water	*E. coli*
	Spike Moss	*S. involvens*	Petroleum ether/benzene/methanol/water	*Pseudomonas* spp.
	Spike Moss	*Selaginella inaequalifolia*	Petroleum ether/benzene/methanol/water	*E. coli*
	Spike Moss	*S. inaequalifolia*	Petroleum ether/benzene/methanol/water	*Pseudomonas* spp.
[40]	Chestnut	*Castanea sativa*	Water	*E. coli*
	Chestnut	*C. sativa*	Water	*Klebsiella pneumoniae*
	Chestnut	*C. sativa*	Water	*S. aureus*
	Chestnut	*C. sativa*	Water	*E. faecalis*
	Clove	*S. aromaticum*	Water	*E. coli*
	Clove	*S. aromaticum*	Water	*K. pneumoniae*
	Clove	*S. aromaticum*	Water	*S. aureus*
	Clove	*S. aromaticum*	Water	*E. faecalis*
[41]	Stonebreaker	*Phyllantus niruri*	Methanol	*E. coli*
[36]	Ketapang	*Terminalia cattapa*	Methanol	*E. coli*
[42]	Chrysanthemum	*Dendranthema grandiflora*	Water	*S.* Derby
	Garlic	*Allium sativum*	Water	*S.* Derby
	Rosemary	*R. officinalis*	Water	*S.* Derby
	Turmeric	*Curcuma longa*	Water	*S.* Heidelberg
	Ginger	*Z. officinale*	Water	*S.* Heidelberg
	Chrysanthemum	*D. grandiflora*	Water	*S.* Heidelberg
	Garlic	*A. sativum*	Water	*S.* Heidelberg
	Chrysanthemum	*D. grandiflora*	Water	*S.* Cubana
	Garlic	*A. sativum*	Water	*S.* Cubana
	Chrysanthemum	*D. grandiflora*	Water	*S.* Orion
	Garlic	*A. sativum*	Water	*S.* Orion
	Chrysanthemum	*C. citratus*	Water	*S.* Enteritidis
	Garlic	*A. sativum*	Water	*S.* Enteritidis
	Chrysanthemum	*D. grandiflora*	Water	*S.* Enteritidis
	Garlic	*A. sativum*	Water	*S.* Infantis
	Chrysanthemum	*D. grandiflora*	Water	*S.* Infantis
	Turmeric	*C. longa*	Water	*S.* Mbandaka
	Garlic	*A. sativum*	Water	*S.* Mbandaka
	Chrysanthemum	*D. grandiflora*	Water	*S.* Mbandaka
	Onion	*Allium cepa*	Water	*S.* Agona
	Garlic	*A. sativum*	Water	*S.* Agona
	Chrysanthemum	*D. grandiflora*	Water	*S.* Agona
	Turmeric	*C. longa*	Water	*S.* Lexington
	Onion	*A. cepa*	Water	*S.* Lexington
	Rue	*Ruta graveolens*	Water	*S.* Lexington
	Garlic	*A. sativum*	Water	*S.* Lexington
	Chrysanthemum	*D. grandiflora*	Water	*S.* Lexington
	Lemongrass	*C. citratus*	Water	*S.* Give
	Garlic	*A. sativum*	Water	*S.* Give
	Chrysanthemum	*D. grandiflora*	Water	*S.* Give
	Garlic	*A. sativum*	Water	*S.* Newport
	Chrysanthemum	*D. grandiflora*	Water	*S.* Newport
	Ginger	*Z. officinale*	Water	*S.* Montevideo
	Garlic	*A. sativum*	Water	*S.* Montevideo
	Lemongrass	*C. citratus*	Water	*S.* Kentucky
	Onion	*A. cepa*	Water	*S.* Kentucky
	Garlic	*A. sativum*	Water	*S.* Kentucky
	Chrysanthemum	*D. grandiflora*	Water	*S.* Kentucky
[43]	Clove	*S. aromaticum*	Ethanol	*E. coli*
	Peppermint	*Mentha piperita*	Ethanol	*E. coli*
	Cinnamon	*Cinnamomum zeylanicum*	Ethanol	*E. coli*
	Coriander	*Coriandrum sativum*	Ethanol	*E. coli*
	Black seed	*Nigella sativa*	Ethanol	*E. coli*
	Garlic	*A. sativum*	Ethanol	*E. coli*

**Table 3:** Application of antibacterial plant extracts in poultry using different management techniques.

Origin of the Extract	Extract Application Route	Extract Administration Responses	Reference
**Antibacterial management before hatching**			
*Q. infectoria*, *P. granatum* and *S. alexandrina*	Eggshell sanitization	At concentrations of 175, 200, and 200 μg/mL, it completely reduces the load of *S.* Typhimurium in the eggshell immersed for 4, 4, and 5 min, respectively. At concentrations of 150, 175, and 200 μg/mL, it completely reduces the load of *S.* Enteritidis in the eggshell immersed for 3, 4, and 4 min, respectively.	[47]
*P. granatum*	Eggshell sanitization	In the proportion of 5 mL/100 mL: Improves hatchability percentage, possibly due to reduced eggshell bacterial load.	[48]
Eucalyptus	Eggshell sanitization	At a concentration of 0.5 and 1%: Reduces the number of *Escherichia coli* colonies on the eggshell. Increases hatching and survival rates of poultry.	[7]
*T. vulgaris*, *C. cassia* and *S. aromaticum*	In ovo	The administration of 0.1 mL of the solution (100 g/400 mL) into the egg (10th day) has positive effects on chick weight and post-hatch performance, in addition to positively influencing the physiological, immunological status, and antioxidant from hatched broiler chickens.	[49]
*M. oleifera*	In ovo	In ovo administration of 0.5 μg/mL of solution improves hatchability rate, day-old chicken weight, and navel area aspects through optimising the yolk components.	[9]
**Antibacterial management after hatching**			
*P. guajava*	Feed	Reduction in avian mortality at concentrations of 3.5% and 4.5%. No change in production performance. In vitro inhibitory effect observed from a concentration of 25 mg/mL on the growth of *Escherichia coli*, *Staphylococcus* spp. and *Streptococcus* spp. isolated from the navel of day-old chicks.	[22]
*C. oleífera*	Feed	At concentrations of 250 and 500 mg/kg: Increases bacterial sensitivity to antibiotics. Improves chick immunity. Inhibits the growth of *Escherichia coli* and *S. aureus* both in vitro and in chicks.	[8]
*C. sativa* + *S. aromaticum*	Water	At the dose of 5 mL/L: Reduction in chick mortality in the first week of rearing probably related to the natural stabilization of bacterial flora and increased immunity, resulting in a reduction in infectious processes. Increases chick weight gain.	[40]
*T. cordifolia*	Feed	At a concentration of 1 g/kg: Reduction in the severity and duration of clinical signs in poultry such as anorexia, listlessness, ruffled feathers, closing of eyes, outstretching and drooping of wings, respiratory distress, and diarrhea. Reduces mortality and *Escherichia coli* load as well as improves post-hatch growth.	[50]
*A. indica*	Water	At a concentration of 10%, it increases the humoral immune response of poultry against *Escherichia coli*.	[10]

## Data Availability

Not applicable.
